# Assessment of Aesthetic Smile Components in Patients With Skeletal Class II Vertical and Horizontal Growth Patterns Compared to Class I Cases

**DOI:** 10.7759/cureus.60399

**Published:** 2024-05-16

**Authors:** Ruchika Pandey, Ranjit Kamble, Dhwani Suchak, Harikishan Kanani

**Affiliations:** 1 Orthodontics and Dentofacial Orthopedics, Sharad Pawar Dental College and Hospital, Datta Meghe Institute of Higher Education and Research, Wardha, IND; 2 Pediatric Dentistry, Sharad Pawar Dental College and Hospital, Datta Meghe Institute of Higher Education and Research, Wardha, IND

**Keywords:** orthodontics, smile design, malocclusion, smile components, balanced smile

## Abstract

Introduction

In orthodontics, having a beautiful smile is very important. It is frequently the main driving force behind people's efforts to enhance their oral health and professional opportunities. Orthodontic and dental treatment planning might benefit greatly when evaluating the aesthetic components of a patient's smile in individuals with varying skeletal growth patterns. In order to help orthodontists achieve the best possible functional and aesthetic results for their patients, the eight elements of a balanced smile are essential to orthodontic therapy. This study aims to evaluate, in comparison to Skeletal Class I Average instances, eight balanced smile components in patients with Skeletal Class II Vertical and Horizontal growth patterns.

Methodology

A total of 45 patients aged 14-30 were selected from the Orthodontics and Dentofacial Orthopedics Out-Patient Department (OPD). They were divided into three groups: Skeletal Class I Average, Skeletal Class II Vertical, and Skeletal Class II Horizontal cases based on their malocclusion type. Patients were made to smile in response to a joke or social conversation and their photos were analyzed using Photopea software (Photopea Inc., Prague, Czech Republic) to determine the eight components of a balanced smile.

Result

Three skeletal classes' worth of smile components were examined in this study. Lip line measurements varied greatly; the highest mean measurement was found in the Skeletal Class II Vertical group (p-value < 0.01). There were no noteworthy correlations found between smile arc and upper lip curvature. Measurements of lateral negative space did not show any significant group differences. On the other hand, a significant correlation was seen in smile symmetry, where asymmetrical smiles were more prevalent in Skeletal Classes I and II Vertical groups (p-value is 0.00072). While arch symmetry suggested a potential relationship between the groups, dental midline alignment revealed possible associations.

Conclusion

Assessing the aesthetic components of smiles in patients with varying skeletal growth patterns, i.e., contrasting Class II Vertical and Horizontal growth patterns with Class I Average cases, provides valuable information about the connection between smile aesthetics and facial skeletal structure. The results suggest that when compared to Class I typical instances, Skeletal Class II Vertical and Horizontal growth patterns may show clear variations in certain aspects of an attractive smile. Comprehending these variations is essential for devising treatment strategies for patients, and additional investigation is needed. In order to obtain optimal aesthetic outcomes, treatment strategies should strive to optimize smile aesthetics while addressing personalized treatment plans that take into account the patient's unique facial features, smiling preferences, and functional requirements.

## Introduction

Synergistic facial muscles work together to produce the most complex expression of the face - a smile. Considering the patient's perspective, this is one of the most significant facial functions. It is among the most important facial expressions, conveying joy, happiness, attitude, and thankfulness [1].

One of the frequent stipulations for treatment in orthodontics is to achieve the optimal aesthetic appearance and swamp the psychological and social dilemma caused by dental anomalies. Smile attractiveness is a crucial issue in orthodontics and frequently the decisive motivating factor for improving career and dental health [1,2]. As patients place more emphasis on the aesthetics of their smiles, it has become increasingly important to consider the soft tissue profile. Smile attractiveness is a crucial factor in orthodontics due to dental anomalies and is often the biggest motivation for improving dental health and career. Numerous studies have been conducted in the literature on the grin's hard and soft tissue aspects. As a result, smile analysis is a crucial stage in the diagnosis, planning, execution, and outcome of any dental procedure aimed at improving appearance. To achieve uniformity of shape in orthodontic treatments, it is necessary to assess the inherent features of the smile. This requires identifying the elements and circumstances that influence these traits [3].

Numerous writers have distinguished between various sorts of smiles; one writer distinguished between two fundamental forms of smiles: Put on a grin (the social grin): it's a deliberate, relaxed, motionless facial expression with only mildly contracted muscles. This is the smile that people usually use to say hello [3]. A person who consistently and voluntarily smiles in social situations is said to be wearing a social smile. It is usually used for greeting, is unopposed and stable, and is produced by a slight contraction of the perioral facial muscles, occasionally revealing the gums [1]. The Duchene smile (maximum smile) or an emotional smile: the term "smile" can be categorized into two types: the social grin, which is a deliberate and relaxed facial expression with mildly contracted muscles, and the genuine smile [3]. The emotional grin, on the other hand, is an involuntary, spontaneous smile brought on by emotional variables like delight. It can be described in various ways, including laughter, tears, knowledge, or disinterest, and the muscles of the face regulate it [1]. Many writers have differentiated between different types of smiles. One author distinguished between two primary forms of smiles: the social smile is a deliberate, relaxed, and motionless facial expression with mildly contracted muscles. This is the smile that people commonly use to greet one another [4].

In orthodontic therapy, Hulsey was the first researcher to measure the correspondence between the location of the lips and teeth when smiling. He concluded that an appealing smile has a smile line ratio near 1.00 based on his research on smile aesthetics [5]. The measurements of upper lip length, upper lip curvature, buccal corridor ratio, smile line ratio, and smile symmetry ratio were used to calculate this ratio. Furthermore, when smiling, the incisal margins of the upper incisors and canines should line up in a curve parallel to the lower lip, according to Sarver's description of the ideal smile arc [6].

In the last 10 years, smile design and analysis have become crucial for orthodontic evaluation and treatment planning. Modern technology allows physicians to measure dynamic lip-tooth connections and incorporate that data into biomechanical and orthodontic problem lists. Digital videography is very helpful for analyzing smiles and facilitating interactions between doctors and patients. The clinical outcome of treatment is influenced by several factors, including the patient's soft-tissue procedure limitations and how much orthodontic or interdisciplinary treatment can align with the patient's and orthodontist's aesthetic objectives [7].

Numerous studies have been published on the aesthetics of the smile. Some studies have analyzed the relationship between lips and teeth using quantitative or quantified measurements. Other studies have assessed smiles using a subjective aesthetic evaluation in which reviewers were asked to rate the pleasingness of participants' smiles [6]. The assessment of components of a balanced smile on various forms of malocclusion for clinical usage has yet to be studied in orthodontics. Thus, this study aimed to statistically assess the relationship between the multiple forms of malocclusions and the aesthetic components of smiles.

Aim

The study aims to assess eight balanced smile components in patients of Skeletal Class II Vertical and Horizontal growth patterns compared to Class I Average cases.

Objective

The primary objectives of this study are to assess the components of a balanced smile in Skeletal Class I Average, Skeletal Class II Vertical, and Skeletal Class II Horizontal patients and compare the components of a balanced smile in Skeletal Class I, Class II Vertical, and Class II Horizontal patients.

## Materials and methods

A total of 45 patients between the ages of 14 and 30 were chosen from the OPD. They were divided into three groups of 15 patients: Skeletal Class I, Class II Vertical, and Class II Horizontal Malocclusion.

The study included participants aged 14 to 30 who were classified as skeletons in Classes I or II. All permanent teeth, apart from the third molars (wisdom teeth), must be present for eligibility. They should also have symmetrical facial features and no prior history of facial trauma. This selection criteria aims to create a homogeneous study group for examining orthodontic outcomes within specific age and skeletal classifications while avoiding confounding characteristics such as dental and facial symmetry.

This study's exclusion criteria involve patients exhibiting specific characteristics that may affect the study outcomes. Patients with a Class III molar or canine relationship are excluded, as are those in the mixed dentition stage. Additionally, individuals who have undergone previous or are currently undergoing orthodontic treatment, or who have cleft lip or palate conditions, are excluded from the study. The study did not include patients with severe dentofacial abnormalities or those lacking teeth. These criteria help ensure a more homogeneous study group focused explicitly on Skeletal Class I and Class II patients, without complicating factors that could impact the evaluation of treatment outcomes.

Methods

An on-the-spot clinical examination was performed on each volunteer patient after they had signed an informed consent form. Patients are classified into three groups: 15 in Skeletal Class I Average, Class II Vertical, and Class II Horizontal Malocclusion based on ANB differentials (Class I if the ANB angle is between 0 and 2 degrees, Class II if >2 degrees, and Class III if <0 degrees), β angle (Class I = 27 to 35 degrees and Class II = <27 degrees), and mandibular plane angle (Average cases = 28-34 degrees, Vertical = >34 degrees, and Horizontal = <28 degrees).

Preparation for photographic standardization steps

The photography environment setup will remain the same in every session. The identical chair height, fixed distance (two feet) between the subject's head and the camera lens, and background are used in all the photographs. To shoot the pictures, a professional digital camera was positioned vertically above a tripod perpendicular to the ground with a magnification of 2x. The Frankfort Horizontal (FH) plane and the interpupillary line were kept parallel to the ground when the subjects were arranged.

After making these preparations, each subject was instructed to relax to capture an image of their frontal rest position. Afterward, they will be urged to interact socially or tell a joke to induce an unprompted smile.

Analysis

All photos are downloaded to a laptop and stored as JPEG/PNG (joint photographic experts group/portable network graphics) images with each file size of around 10 megabytes. The photos were loaded into Photopea (Photopea Inc., Prague, Czech Republic), an online photo editing software, to finish the study. Then, measurements were calibrated using the software's unit tool, and eight components of a balanced smile were determined and compared Skeletal Class II Vertical and Class II Horizontal Malocclusion against Skeletal Class I Average.

Sample size calculation

Formula using mean difference

n1 = n2 = 2(Zα + Zβ)2σ2)/d2 ​​​​

Zα = 1.96, α = Type I error at 5%

Zβ = 1.28 at equivalence (1 - β) = Power at 90%

σ = standard deviation

Primary variable height of commissure in rest position

(Height of commissure in rest position in Class II Horizontal growth pattern of the face) Mean ± SD = 31.27 ± 4.26 (as per reference article)

(Height of commissure in rest position in Class II Vertical growth pattern of the face) Mean ± SD = 25.23 ± 5.79 (as per reference article)

Difference = (31.27 - 25.23) = 6.04

Pooled SD = (5.79 + 4.26)/2 = 5.025 (as per reference articles)

N1 = 2*[(1.96 + 1.28)2(5.025)2]/(6.04)2 = 15

Total samples required = 15 per group = 45

Study reference: Kabalan et al. [1].

Statistical analysis

A one-way analysis of variance (ANOVA) test has been done for inter-group comparison to determine the significance between the control and test groups. A Chi-square analysis determines the association between the variables at a significant value of p < 0.05.

## Results

The study comprises three groups: Group 1 (Skeletal Class I Average), Group 2 (Skeletal Class II Vertical), and Group 3 (Skeletal Class II Horizontal). The eight elements of a balanced smile were used as parameters to analyze smiles in these groups: line, smile arc, curvature of the upper lip, lateral negative space (buccal corridor), symmetry of the smile, frontal occlusal plane, dental, and gingival components constitute a few of these characteristics. By examining these specific factors, researchers wanted to thoroughly analyze and compare smile characteristics across different skeletal classifications, providing valuable information on smile aesthetics in orthodontic conditions.

The data presents measurements of the lip line for three different groups and the total. Group 1 has a mean of 25.0667, Group 2 has a mean of 37.3333, and Group 3 has a mean of 21.2667. The statistical analysis suggests significant differences between the groups. Group 2 has the highest mean lip line measurement (37.3333), followed by Group 1 (25.0667) and then Group 3 (21.2667) (Table 1).

**Table 1:** The data presents measurements of lip lines for three different groups and the total. A p-value of less than or equal to 0.05 is considered significant. So, p-value = <0.01 shows a statistically significant result.

Lip line	N	Mean	Standard deviation	Standard error	95% confidence interval for mean		Minimum	Maximum	F-value	p-value
					Lower bound	Upper bound				
1.00	15	25.0667	6.32982	1.63435	21.5613	28.5720	16.00	38.00	12.57933	<0.01
2.00	15	37.3333	13.55237	3.49921	29.8283	44.8384	16.00	65.00		
3.00	15	21.2667	5.33809	1.37829	18.3105	24.2228	15.00	30.00		
Total	45	27.8889	11.32821	1.68871	24.4855	31.2923	15.00	65.00		

The statistical information on the distribution of smile arcs is examined across three groups; the frequencies and percentages for consonant and non-consonant smile arc categories are provided within each group. The chi-square test indicates that there may not be a significant association between smile arc and consonant type overall, as suggested by the p-value (probability value) of 0.5365, higher than the typical significance level of 0.05 (Table 2).

**Table 2:** The data presents the distribution of smile arc for three different groups and the total. A p-value of less than or equal to 0.05 is considered significant. So, p-value = 0.5365 shows a statistically insignificant result.

			Group			Total	Chi-square	p-value
			1	2	3			
Smile arc	Consonant	Frequency	6	7	9	22	1.245	0.5365
		%	40.0%	46.7%	60.0%	48.9%		
	Non-consonant	Frequency	9	8	6	23		
		%	60.0%	53.3%	40.0%	51.1%		
Total		Frequency	15	15	15	45		
		%	100.0%	100.0%	100.0%	100.0%		

The relationship between upper lip curvature across three groups presents frequencies and percentages for each combination, along with Chi-square statistics given in the table. The results suggest no significant association between upper lip curvature and the specified categories, as indicated by the p-value of 0.66 (Table 3).

**Table 3:** The relationship between upper lip curvature across three groups presents frequencies and percentages for each combination, along with Chi-square statistics. A p-value of less than or equal to 0.05 is considered significant. So, p-value = 0.66 shows a statistically insignificant result.

			Group			Total	Chi-square	p-value
			1	2	3			
Downward	Frequency	6		7	5	18	2.41	0.66
	%	40.0%		46.7%	33.3%	40.0%		
Straight	Frequency	9		8	9	26		
	%	60.0%		53.3%	60.0%	57.8%		
Upward	Frequency	0		0	1	1		
	%	0.0%		0.0%	6.7%	2.2%		
Total		Frequency	15	15	15	45		
		%	100.0%	100.0%	100.0%	100.0%		

The statistical analysis suggests that there is no significant difference in lateral negative space measurements between the groups, as indicated by the F-value of 0.1556 and a corresponding p-value of 0.856 for the average group (Table 4).

**Table 4:** The statistical analysis of lateral negative space measurements between the groups. A p-value of less than or equal to 0.05 is considered significant. So, p-value = 0.856 shows a statistically insignificant result.

Lateral negative space	N	Mean	Standard deviation	Standard error	95% confidence interval for mean (lower bound)	95% confidence interval for mean (upper bound)	Minimum	Maximum	F-value	p-value
1.00	15	26.2000	5.26715	1.35997	23.2832	29.1168	17.00	38.00	0.1556	0.856
2.00	15	25.3333	8.53285	2.20317	20.6080	30.0587	16.00	45.00		
3.00	15	24.8667	5.64253	1.45689	21.7419	27.9914	17.00	32.00		
Total	45	25.4667	6.51432	0.97110	23.5095	27.4238	16.00	45.00		

The table explores the relationship between smile symmetry and three categories. The table provides frequencies and percentages for each combination, along with Chi-square statistics. The analysis reveals a highly significant association between smile symmetry and the specified categories. The asymmetrical smile is more common in categories of average and vertical, while the symmetrical smile predominates in horizontal (Table 5). The analysis of the possible correlation between the frontal occlusal plane and three categories was seen, but the results showed no significant association (Table 6).

**Table 5:** Explores the relationship between smile symmetry and three categories. A p-value of less than or equal to 0.05 is considered significant. So, p-value = 0.00072 shows a statistically significant result.

			Group			Total	Chi-square	p-value
			1	2	3			
Smile symmetry	Asymmetrical	Frequency	11	11	2	24	14.464	0.00072
		%	73.3%	73.3%	13.3%	53.3%		
	Symmetrical	Frequency	4	4	13	21		
		%	26.7%	26.7%	86.7%	46.7%		
Total		Frequency	15	15	15	45		
		%	100.0%	100.0%	100.0%	100.0%		

**Table 6:** Analyzed the possible correlation between the frontal occlusal plane and three categories, but the results showed no significant association. A p-value of less than or equal to 0.05 is considered significant. So, p-value = 0.914 shows a statistically insignificant result.

			Group			Total	Chi-square	p-value
			1	2	3			
Frontal occlusal plane	Cant	Frequency	7	8	8	23	0.178	0.914
		%	46.7%	53.3%	53.3%	51.1%		
	No cant	Frequency	8	7	7	22		
		%	53.3%	46.7%	46.7%	48.9%		
Total		Frequency	15	15	15	45		
		%	100.0%	100.0%	100.0%	100.0%		

The first six elements of the smile concentrate on how the lips and surrounding soft tissues frame the grin, as well as how the teeth and lips interact. The quality and attractiveness of the dental elements that a smile displays, as well as their flawless integration, all contribute to its overall pleasing appearance. The size, shape, color, and alignment of the teeth as well as the symmetry of the arch and the center line alignment all contribute to the overall appearance of a smile. To create a smile that is harmonious and aesthetically beautiful, all of these dental qualities are essential.

Teeth differ in size, shape, alignment, color, and tip depending on environment, development, and heredity. The appearance of our teeth, including their size, form, alignment, and color, is influenced by both environmental and genetic influences. Genetics determines the basic chemistry and morphology of teeth; nevertheless, lifestyle choices, nutrition, and dental hygiene habits impact tooth color, alignment, and overall oral health. Dental treatments, including orthodontics, restorative, and cosmetic dentistry, can potentially have an impact on teeth. Individual differences in dental morphology are caused by the interaction of hereditary and environmental factors. An efficient way to distinguish between variations in three distinct groups is to evaluate the arch symmetry and midline alignment.

The analysis of the potential relationship between the three groups and the dental midline yielded a borderline p-value of 0.053 and a Chi-square statistic of 5.85 (Table 7).

**Table 7:** The analysis of the potential relationship between the three groups and the dental midline. A p-value of less than or equal to 0.05 is considered significant. So, p-value = 0.053 shows a statistically insignificant result.

			Group			Total	Chi-square	p-value
			1	2	3			
Dental midline	Matching	Frequency	14	11	15	40	5.85	0.053
		%	93.3%	73.3%	100.0%	88.9%		
	Not matching	Frequency	1	4	0	5		
		%	6.7%	26.7%	0.0%	11.1%		
Total		Frequency	15	15	15	45		
		%	100.0%	100.0%	100.0%	100.0%		

The correlation between arch symmetry and three groups, with a Chi-square score of 4.447, indicated a possible relationship. The p-value of 0.1082, however, is higher than the customary significance limit of 0.05, suggesting that the link may not be statistically significant (Table 8).

**Table 8:** The correlation between arch symmetry and the three groups, along with a Chi-square score, indicated a possible relationship. A p-value of less than or equal to 0.05 is considered significant. So, p-value = 0.1082 shows a statistically insignificant result.

			Group			Total	Chi-square	p-value
			1	2	3			
Arch symmetry	Asymmetrical	Frequency	9	9	4	22	4.447	0.1082
		%	60.0%	60.0%	26.7%	48.9%		
	Symmetrical	Frequency	6	6	11	23		
		%	40.0%	40.0%	73.3%	51.1%		
Total		Frequency	15	15	15	45		
		%	100.0%	100.0%	100.0%	100.0%		

Gingiva has a variety of properties that influence how they appear in a smile, including color, form, texture, and height. Inflammation, irregular gingival margins, open embrasures, and blunt gingival tissue between teeth can all have a detrimental effect on the smile's overall appearance. It's important to keep in mind that these conditions can be impacted by a range of factors and are not always prevalent in certain types of malocclusions. Oral hygiene habits, genetic predispositions, dental treatments, and the existence of underlying periodontal or dental diseases are some of the variables that can affect these traits. Therefore, it cannot be conclusively determined that a particular type of gingival health is predominant in a specific malocclusion.

## Discussion

In orthodontic practice, the emphasis on aesthetics has traditionally been on enhancing the profile, overlooking the smile's frontal view [8]. An attractive smile is essential, as it can significantly impact a person's self-confidence, social interactions, and overall well-being. Orthodontic treatments should focus on correcting dental misalignments and enhancing smile aesthetics, to ensure patients have a harmonious and appealing smile [9]. Smiles that are aesthetically pleasing contribute to positive perceptions of facial attractiveness, which can profoundly affect an individual's personal and professional life. Orthodontists strive to achieve functional occlusions and aesthetically pleasing smiles, recognizing the significant impact of a confident smile on a person's quality of life [10,11].

While obtaining a functional occlusion is crucial for orthodontic treatment, patients are beginning to place more value on aesthetic results, like a radiant smile and a young-looking face [12,13]. Although occlusal criteria have historically been the focus of orthodontists, patients want more than just alignment. Understanding the fundamentals of the perfect smile is essential for efficient treatment planning [14,15]. Some of these principles include incisal design, central incisor ratios and symmetry, spacing, gingival presentation, buccal corridors, and tooth details. Orthodontists can optimize smile aesthetics and occlusal function by incorporating these ideas into diagnosis and treatment programs. Clinical examples show the importance of incisal design and central incisor dimensions to achieving aesthetically acceptable results. Consequently, for orthodontic treatment to be effective and successful, a comprehensive plan that considers both occlusal and aesthetic criteria is essential [16,17].

This study emphasizes the importance of considering all relevant variables when diagnosing and planning treatment. It examines eight essential components of the smile, including the alignment of the lip line with the gingival border and lip elevation, which is affected by factors such as age and gender. Essential factors include the curvature of the upper lip, lateral negative space, frontal occlusal plane, smile symmetry, dental components (such as tooth size, form, and alignment), and gingival components (like color and contour). Understanding each element's role in the overall aesthetic appeal can help orthodontic interventions produce balanced, harmonious smiles, which patients are increasingly cherishing.

Several studies have been done before that emphasize the importance of smile aesthetics in dental or skeletal malocclusion. Tarnach et al. used β angle, ANB angle, and Wits appraisal to skeletally divide 60 individuals between the ages of 17 and 25 into Groups I and II. Various parameters were measured on cephalograms, and Adobe Photoshop was used to quantify smiles on facial photographs of patients with Class II. Two malocclusions had the longest upper lips, whereas Class I malocclusion patients had the shortest upper lips. The maxillary incisal show was the most incredible in Class II Div 1 malocclusion patients and lowest in Class II Div 2 malocclusion patients when they were at rest and smiling. Different skeletal patterns display their distinctive grin qualities. When planning orthodontic treatment, the relationship between skeletal and dental impacts on the smile's aesthetics should be considered [18]. Kakadiya et al. included 77 individuals with an average age of 18 years in a study. Each patient's digital camera stream was edited to isolate a single picture representing the patient's staged social grin, and that image was then saved. A vernier caliper was used to analyze each image and calculate the smile score. Class II Div 1, Class II Div 2, and Class III did not significantly vary in the left and right buccal corridor space on the smile; the Class I patient was the only one with a significant difference. The first premolar in Class I, Class II Div 1, Class II Div 2, and Class III was the last maxillary tooth that was most commonly visible [19]. Cheng et al. evaluated smile aesthetic variables using facial photographs of 106 patients, while hard-tissue characteristics were assessed using lateral cephalographic tracings. Based on their overjet, the patients were split into three categories. Before receiving orthodontic treatment, all smile measurements substantially varied between the three groups, except the upper midline and buccal corridor ratios [20]. Ahmad et al. aimed to use standardized photographic analysis to evaluate the smile parameters in various dentoalveolar malocclusion instances [21]. Subjects and procedures: A total of 132 individuals were randomly chosen, ranging in age from 18 to 24. The study is divided into four categories based on angle classification. Each subject had standardized extraoral photos taken of them in the profile, such as social smile, maximum smile, and resting posture. Smile analysis was performed using software to determine specific measurements digitally. The extent of the upper and lower lips changed significantly. When you smile, your lips widen considerably. The smile region didn't reveal any appreciable differences in social smiles between groups. The control group had a mild grin. Class I exhibited a high smile and bimaxillary protrusion, spacing, and open bite subgroups [3]. Kabalan et al. evaluated a sample of 60 patients from three different malocclusion classes: Class I, Class II Div 1, and Class II Div 2. For each patient, an appropriate camera mounted at a proper distance from the photographed face was used to record a video for five to 10 seconds. Two photographs for each plane of the facial still were selected for each subject from the streaming video recording. Some of the examined factors had statistically significant differences. The Class II Div 1 group had mean values for upper lip thickness, commissure height, gum breadth, maxillary incisor show, and inter-labial gap that were higher than those of the other two groups [1].

A study by Nouh et al. compared smile parameters between Class I and Class III male subjects. Class III males smiled more than Class I participants, with more enormous smiles, less incisor exposure, wider smile arcs, and more non-consonant and flat smile arcs; nevertheless, soft tissue measurements at repose revealed no significant differences. Class I subjects showed a more considerable grin breadth and height, while Class III subjects showed a more extensive maxillary incisor presentation. These findings underscore the importance of understanding the differences in smiles across different skeletal classifications to optimize therapeutic outcomes and patient satisfaction [22]. In three age groups of 100 typical Himachali volunteers, Sachdeva et al. used Clever Ruler software to assess the impact of a grin on facial aesthetics. He found the maxillary incisal display decreased by 2.2 mm, the inter-labial gap decreased by 2.0 mm, and the grin index increased by 0.7 mm from younger to older age groups. It may be observed that as people age, their smiles tend to get wider across and narrower vertically [23].

By comprehending how skeletal differences impact smile aesthetics, dentists can successfully customize treatment regimens to maximize both look and function. The selection of suitable orthodontic therapies and related procedures to successfully address aesthetic concerns is guided by such assessments. Orthognathic surgery to adjust skeletal relationships, orthodontic tooth movement to address malocclusions, and dental restorations or gingival contouring to improve smile symmetry and balance are examples of adjunctive therapies. The limitations of this study include small sample numbers and a focus on specific variables, such as age or gender, which may restrict the generalizability of their findings to larger populations. Assessing smile aesthetics frequently requires subjective assessments, which can differ between assessors. Even with the use of established methodologies and software, there may be subjective components in interpreting smile characteristics. This study has limited our ability to establish long-term causal correlations between orthodontic procedures and smile aesthetics. The longitudinal studies would provide stronger evidence of the effect of orthodontic therapy on smile aesthetics.

## Conclusions

Several elements of a balanced smile were analyzed in this study using three different groups: Skeletal Class I Average, Skeletal Class II Vertical, and Skeletal Class II Horizontal. This study's primary focal points are the lip line, smile arc, upper lip curvature, lateral negative space, smile symmetry, frontal occlusal plane, dental components, and gingival components. We analyzed these specific parameters within each skeletal category to comprehensively understand and compare smiling traits. Significant insights into the aesthetics of smiles in orthodontic contexts and unique skeletal patterns were obtained from this study. The lip line measurements varied significantly among groups, with the largest mean measurement found in Skeletal Type II Vertical. No apparent relationships were found between upper lip curvature and the smile arc. Measurements of lateral negative space did not show any significant group differences. On the other hand, a significant correlation was seen in smile symmetry, where asymmetrical grins were more prevalent in Skeletal Classes I and II Vertical groups. While arch symmetry suggested a probable relationship between the groups, dental midline alignment revealed possible relationships. Overall, a smile's aesthetic appeal is influenced by several characteristics, including gingival appearance, tooth size, shape, color, and alignment. However, because these factors depend on one another, their impact may differ between skeletal classifications.
